# The Role of Nucleotide Excision by Reverse Transcriptase in HIV Drug Resistance

**DOI:** 10.3390/v2020372

**Published:** 2010-01-28

**Authors:** Antonio J. Acosta-Hoyos, Walter A. Scott

**Affiliations:** Department of Biochemistry and Molecular Biology, University of Miami Miller School of Medicine, P.O. Box 016129, Miami, FL 33101-6129, USA; E-Mail: AAcosta3@med.miami.edu (A.J.A.-H.)

**Keywords:** reverse transcriptase, HIV, antiviral drug resistance, AZT resistance, nucleotide excision, NRTIs, suppression of drug resistance, foscarnet

## Abstract

Nucleoside reverse transcriptase (RT) inhibitors of HIV block viral replication through the ability of HIV RT to incorporate chain-terminating nucleotide analogs during viral DNA synthesis. Once incorporated, the chain-terminating residue must be removed before DNA synthesis can continue. Removal can be accomplished by the excision activity of HIV RT, which catalyzes the transfer of the 3′-terminal residue on the blocked DNA chain to an acceptor substrate, probably ATP in most infected cells. Mutations of RT that enhance excision activity are the most common cause of resistance to 3′-azido-3′-deoxythymidine (AZT) and exhibit low-level cross-resistance to most other nucleoside RT inhibitors. The resistance to AZT is suppressed by a number of additional mutations in RT, most of which were identified because they conferred resistance to other RT inhibitors. Here we review current understanding of the biochemical mechanisms responsible for increased or decreased excision activity due to these mutations.

## 1. Introduction

Reverse transcriptase (RT) is the replication enzyme for HIV and a major target for antiretroviral drug development. The enzyme contains DNA polymerase and ribonuclease H (RNase H) activities catalyzed by two different active sites within the heterodimeric enzyme structure. Viral DNA synthesis is initiated from a tRNA molecule annealed to the viral RNA genome copying the viral genome until a complementary (minus-strand) DNA molecule is formed. The RNase H activity plays an important role by cleaving the genomic RNA into fragments and further degrading these fragments until they are released from the minus-strand DNA. Viral nucleocapsid protein (NC protein), which accompanies the viral genome during infection, is also a participant in this process and facilitates the displacement of annealed RNA fragments through its helix destabilizing activity. Specific RNA fragments (characterized by the presence of a polypurine tract (PPT) sequence) preferentially escape displacement or degradation and serve as primers for initiation of second strand (plus-strand) DNA synthesis at two sites on the genome of HIV. Plus-strand synthesis results in a double-stranded product that serves as substrate for integration into the host cell chromosomes forming an integrated provirus from which viral messenger RNAs and viral genomes are transcribed.

An effective way to inhibit HIV replication is to introduce a nucleoside analog that can be activated to the triphosphate form by cellular kinases and is recognized as a substrate for DNA chain elongation by HIV RT but lacks a 3′OH, so that incorporation into the viral DNA leads to chain termination. Drugs that inhibit HIV replication by this mechanism are termed nucleoside RT inhibitors (NRTIs). The founding member of this class of drugs was β-d-(+)-3′-azido-3′-deoxythymidine (AZT or zidovudine), which was approved by the U.S. Food and Drug Administration for treatment of HIV infection in 1987 [1]. Seven other NRTIs have been approved by the FDA as of the end of 2009 [Table 1], and several additional NRTIs are currently in pre-clinical and clinical stages of development. Another class of RT inhibitors (non-nucleoside RT inhibitors, NNRTIs) acts by binding to a separate site on the enzyme and allosterically inhibiting DNA synthesis. NNRTIs are discussed in several recent reviews [2–9] and will not be considered further here. An additional class of non-nucleoside RT inhibitors (nucleotide-competing RT inhibitors; NcRTIs) has been recently described that competes with nucleotide incorporation but does not lead to chain termination. NcRTIs are the subject of a separate review in this special issue of *Viruses* [10]. The pyrophosphate (PPi) analog, phosphonoformic acid (foscarnet, PFA), inhibits RT by a different mechanism, and there has been recent interest in identifying additional PPi-analog inhibitors [11–13].

Chronic therapy with NRTIs leads to selection of drug-resistance mutations and loss of the effectiveness of the therapy. Characteristic RT mutations are usually selected by each NRTI (shown in Table 1) and combination therapies may select yet different combinations of mutations. Mutations that confer NRTI resistance fall into two classes. Some result in an altered enzyme that has increased ability to discriminate against the incorporation of the analog in favor of the natural dNTP substrate (resistance mechanism identified as “discrimination” in Table 1). Other mutations confer an increased ability of RT to remove the chain-terminating nucleotide after it has been incorporated (resistance mechanism identified as “excision” in Table 1). This review focuses specifically on the role of the excision mechanism in HIV drug resistance. Excision is the major mechanism of HIV-1 resistance to AZT [6,9,14–18].

## 2. Mutants of HIV-1 RT that Enhance the Rate of Nucleotide Excision

AZT-resistant strains of HIV-1 were initially shown to contain specific mutations mapping to the N-terminal portion of the RT coding region [20]. Further studies have identified mutations at six codons in RT that are associated with high-level AZT resistance: M41L, D67N, K70R, L210W, T215F/Y, and K219Q/E [21–23]. These mutations are also selected by d4T therapy [for reviews, see Refs. 14,24], which led to them being named thymidine analog resistant mutants (TAMs). They also confer low-level cross-resistance to most other NRTIs [25]. Other names have been applied to these mutations (including NAMs, NEMs, and EEMs) to reflect their broad effect across NRTIs; however, the term “TAMs” is the most widely used. The TAMs appear sequentially during AZT therapy, with K70R and T215F or Y appearing first [26,27]. Association between different TAMs in viruses recovered from clinical specimens has indicated two different evolutionary patterns for these mutations. The most common pattern (TAM-1) includes M41L, L210W and T215Y and excludes K70R. The second pattern (TAM-2) includes D67N, K70R, T215F and K219Q/E [for a more detailed discussion, see Ref. [17]).

**Figure 1.**
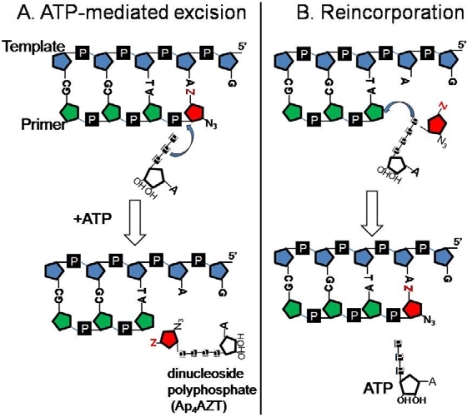
ATP-mediated excision of AZTMP and the use of Ap_4_AZT as substrate for AZTMP incorporation. (A) ATP attacks the phosphodiester bond removing the drug-MP from the primer terminus, forming a dinucleoside polyphosphate and an unblocked primer [32]. (B) Ap_4_AZT is recognized as an analog of AZTTP leading to incorporation of AZTMP and release of ATP [34,35]. The template and primer are shown in blue and green, respectively; AZT is in red.

The molecular mechanism for AZT resistance eluded investigators for several years because the resistance could not be replicated in biochemical assays [20,28–30]. That changed with reports that AZT-resistant RT could excise the chain terminator more efficiently if an acceptor substrate such as PPi [31] or nucleoside triphosphate [32,33] was added to the reaction mixture. PPi-dependent excision (pyrophosphorolysis) is the reversal of dNTP incorporation and is carried out by most DNA polymerases. HIV-1 RT also has the ability to carry out a pyrophosphorolysis-like reaction using nucleoside di- or triphosphates instead of PPi [32]. Figure 1A shows nucleotide-dependent excision with ATP as the acceptor substrate and AZT-terminated primer-template, which produces the dinucleoside tetraphosphate, Ap_4_AZT, as product.

TAM-containing RTs have been variously reported to support either increased or decreased excision with PPi as substrate; however, there is general agreement that ATP-dependent excision is significantly elevated in these mutants. Comparison of ATP-dependent excision activity between various mutant RTs has provided a strong biochemical correlate for AZT resistance measured by infectivity assays over a 150-fold range of excision activities [15,36,37]. ATP is likely to be the most biologically relevant acceptor substrate for the excision reaction based on molecular modeling studies [38,39] and the fact that the concentration of ATP is greater than that of other potential acceptor substrates in most living cells (reviewed in Ref. [40]). PPi accumulates during metabolic stimulation of lymphoid cells [41], and PPi-dependent excision occurs much more rapidly than ATP-dependent excision, suggesting that the PPi-dependent reaction may also play a role in AZT susceptibility under some circumstances [41,42].

Since PPi-dependent excision regenerates the triphosphate form of the chain-terminating nucleotide, it is possible that the excision product will be reincorporated before it can be released from the surface of the enzyme or it may be released and immediately rebinds leading to reincorporation. The same is true for the ATP-dependent excision product, which is also an efficient substrate for reincorporation by TAM-containing RTs (illustrated in Figure 1B)[34,35]. It is often assumed that rebinding and reincorporation of the dinucleoside tetraphosphate will be disfavored in comparison with the triphosphate; but recent studies show that chain termination by Ap_4_ddNs [34] or Ap_4_AZT [35] is as efficient as chain termination by the corresponding triphosphates when tested with TAM-containing RTs. The affinity of Ap_4_AZT for the mutant RT is ∼10-fold greater than AZTTP. Further understanding of the resistance phenotype will benefit from characterization of factors that could influence excision product reincorporation.

While TAMs confer resistance to most NRTIs, resistance is much greater for AZT than for other NRTIs. This selectivity is only seen in biochemical assays when physiological concentrations of dNTPs are added to the reaction mixture [33,43]. After chain termination with an NRTI, RT attempts to add the next complementary dNTP; but, because of the lack of a 3′OH, dNTP binding produces a dead-end complex [33,43,44]. This traps RT in the post-translocation position relative to the primer terminus and excision is inhibited because the catalytic residues are positioned to add the next nucleotide rather than to remove the previous one. More detailed discussion of RT positioning and translocation is given in the next section. While excision of most NRTIs is inhibited when physiological concentrations of dNTPs are present, primer terminated with AZT remains in the pre-translocation position except when high dNTP concentrations are added [45–47]. The presence of AZT on the primer strand may interfere with dNTP binding or the pre-translocation configuration may be stabilized through interactions between the AZTMP-terminated primer and RT. Phenotypic assays used to determine drug susceptibility are typically carried out in cells that have high intracellular dNTP concentrations. Under these conditions, excision of most NRTIs would be inhibited; however, AZT excision is not inhibited and the assays give the appearance of selective AZT resistance. *In vivo*, the selectivity will depend on intracellular dNTP concentrations, which differ greatly between different cell types (for a review, see [40]).

Understanding of the contributions of the individual TAMs to the mechanism of enhanced excision has focused on mutation from threonine to an aromatic residue at codon 215, which is a critical component of the resistance genotype [48]. Modeling of ATP into the RT structure shows that these mutations place the aromatic ring of Y or F in position to form π-π-stacking interactions with the adenine moiety from ATP [38,39]. The efficiency with which alternative nucleoside triphosphates serve as excision acceptors is consistent with their ability to form π-π-stacking interactions with the aromatic residue at position 215 [49].

Excision is also enhanced by short insertions following codon 69 in the fingers domain of RT. These mutations occur only rarely in HIV-1-infected patients but are associated with resistance to multiple NRTIs and high levels of excision activity [50,51]. The mutations are usually dipeptide insertions that occur in combination with a T69S mutation and a variable number of TAMs. Finger-insertion mutants of RT have been the subject of recent reviews [52–54].

HIV RT is versatile in its ability to accommodate modification of its dNTP substrate for DNA synthesis. Analogs of dNTPs bearing bulky hydrophobic groups attached to the γ-phosphate [55–57] as well as amino acid phosphoramidate conjugates with deoxynucleoside monophosphates are readily incorporated by RT [58–61]. To a limited extent, RT is also able to use deoxynucleoside diphosphates for incorporation [62]. The dinucleoside tetraphosphate product of ATP-dependent excision (Ap_4_ddN) can also take part in the incorporation reaction [34,35,63] as illustrated in Figure 1B. Use of Ap_4_ddN as substrate for chain termination is greatly enhanced in RT containing TAMs by comparison with wild-type (WT) RT [34,35]. The enhancing effect of the TAMs is further increased by addition of a 2′(3′)-O-(N-methyanthraniloyl) group to the ribose moiety of the adenosine [34].

The affinity for Ap_4_AZT by AZT-resistant RT is about 30,000 times greater than for ATP and about 10 times greater than for AZTTP [35]. Hydrolysis-resistant analogs of Ap_4_AZT have been proposed as potential novel RT inhibitors to take advantage of this high-affinity interaction [64]. The affinity for ATP in the excision reaction is weak. A range of *K*_d_ from 0.16 to 1.6 mM has been reported for primers terminated with various chain-terminating nucleotides [32,42,65]. The literature is not in agreement as to whether affinity for ATP is higher or lower in RTs containing TAMs than in WT RT [15,38,42,65]; however, the reports agree that both the *k*_cat_ and catalytic efficiency are consistently enhanced by the presence of TAMs. This suggests that the TAMs accelerate an intermediate step that is rate limiting in both the forward and backward reactions. The rate of formation of a reaction intermediate resembling the Ap_4_ddN product may be limited by the ability of ATP to bind in the appropriate orientation to carry out the nucleophilic attack to excise the ddNMP from the primer. The rate of formation of the same intermediate in the reverse reaction would also be limited by the ability of Ap_4_ddN to bind in an orientation that allows nucleophilic attack by the 3′OH on the primer and the same structural features of WT RT that restrict the rate of excision also determine the rate of incorporation. By introducing new interactions with the adenosine moiety of ATP, the TAMs adjust the orientation of the β and γ phosphates of ATP or Ap_4_ddN to increase the rate of excision or incorporation, respectively. The finding that a bulky substituent on the adenosine portion of the dinucleoside tetraphosphate [2′(3′)-*O*-(N-methylanthraniloyl)-Ap_4_ddG] enhances the difference between WT and TAM RT [34] suggests that the increased rate of incorporation is derived from the improved ability of TAM RTs to accommodate the ATP as a leaving group even when it is conjugated to a bulky structure.

## 3. Mutations of HIV-1 RT that Decrease the Rate of Nucleotide Excision and Suppress AZT Resistance

A number of RT mutations have been identified that have reduced excision activity [37]. Most of these mutants were isolated even before the excision activity of RT was recognized because of the fact that they conferred resistance to PFA and enhanced sensitivity to AZT [66–69]. Mutations that confer resistance to PFA are summarized in Table 2.

**Figure 2.**
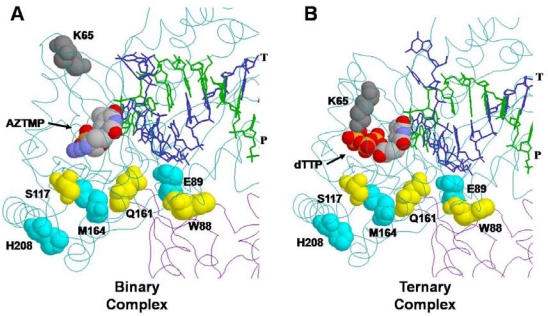
Locations of residues altered by PFA-resistance mutations in the structures of binary and ternary complexes of HIV-1 RT. **(A)** Binary complex of HIV-1 RT with AZTMP-terminated primer-template occupying the dNTP-binding site (N site) [PDB structure 1N6Q, Ref. 45]. **(B)** Ternary complex of HIV-1 RT with ddAMP-terminated primer-template and dTTP occupying the N site [PDB structure 1RTD, Ref. 85]. The template (T) is shown in blue, and the primer strand (P) in green. The structure occupying the N site in each complex is shown as a space-filling model (atoms indicated by CPK color scheme). Residues that are substituted in the indicated PFA-resistant mutants are also shown as space-filling models. The fingers-domain residue K65 is shown in gray. Palm-domain residues are shown in yellow or cyan using contrasting colors to distinguish overlapping structures [37].

The PFA-resistance phenotype is readily explained for mutations in residues that interact with the β or γ- phosphates of the incoming dNTP (e.g., K65 and R72), since they participate directly in the binding of PPi and PFA [85,86]. Reduced excision activity can be explained by reduced or altered binding to the ATP acceptor substrate for excision. K65 interaction with the γ-phosphate of dTTP is illustrated in Figure 2B. Effects of the K65R mutation will be discussed in more detail in the next section. Mutations in the palm region of RT that are located far from the dNTP-binding site (Figure 2) are less easily explained. Mutations at these residues (e.g., W88) conferred the highest level of PFA resistance and were most likely to be selected in PFA-treated patients [68] or by serial passage of virus in culture in the presence of increasing concentrations of PFA [68,67]. Of these mutations, E89K affects a residue that can interact with the template strand. The other residues do not have direct interactions that can explain the PFA resistance. The mechanism of PFA-resistance and reduced excision activity for most of these mutants is unclear.

**Figure 3.**
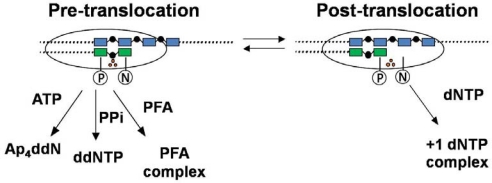
Positioning of RT with respect to the 3′-end of the primer. The pre-translocation complex places the RT active site in position to carry out excision with ATP or PPi as acceptor substrate. The primer terminus occupies the dNTP-binding site (N site) on RT. RT is stabilized in this position by binding PFA. The post-translocated complex places the RT in a position to add the next deoxynucleotide. The primer terminus occupies the primer-binding site (P site) on RT. RT is stabilized in this position by binding the next complementary dNTP [38,45–47,87]. The template and primer are shown in blue and green, respectively. The three orange dots correspond to active site catalytic residues, which are located near the boundary between the N and P sites.

During viral replication RT undergoes a series of conformational changes as the enzyme binds to the P/T substrate as the binary complex (RT·P/T), followed by binding of the dNTP substrate to form the ternary complex (RT·P/T·dNTP), incorporation of dNMP, release of the PPi product and translocation to the next position. The binary complex can exist in two positions [38,45] that have been designated as the pre-translocation complex (where the primer terminus occupies the dNTP binding site or N site) and the post-translocation complex (where the primer terminus occupies a site adjacent to the N site [designated as the P site] leaving the N site open to accept the incoming dNTP) (Figure 3).

When the dNTP binds, a stable ternary complex (RT·P/T·dNTP) is formed [44] and the enzyme is locked in the post-translocation position until phosphodiester bond formation can occur. PFA is also able to form a ternary complex (RT·P/T·PFA) that locks the enzyme in the pre-translocation position with the primer-terminus in the N site [12,37,47,87–89]. The binary complex can oscillate between the forward and backward positions but can only bind dNTP when in the post-translocation position and can only carry out ATP- or PPi-mediated excision when in the pre-translocation position (Figure 3). Sarafianos *et al.* [45] have obtained crystal structures of binary complexes with AZT-terminated primer terminus in either the N-site or the P-site configurations. Several crystal structures of the RT·P/T·dNTP ternary complex have been reported [85,90] including new structures of the K65R mutant ternary complex [86] that will be considered in more detail in the next section.

Marchand *et al*. [88] used site-specific footprinting to show that DNA synthesis by HIV-1 RT is most sensitive to inhibition by PFA at positions on the P/T that bind RT preferentially in the pre-translocational state. Sensitivity to PFA is reduced with sequences that favor the post-translocational state. The E89K mutation destabilized the pre-translocational complex, disfavoring both PFA binding and excision. The crystal structures suggest that direct contact may occur between E89 and the −2 position on the template strand. Altered binding of the E89K mutant RT to the template phosphate backbone could be responsible for destabilizing the pre-translocational binary complex and reducing the affinity for PFA.

In sum, mutations at the PPi-binding site confer PFA resistance by impairing its ability to bind to RT. They confer AZT hypersensitivity and suppress AZT resistance due to TAMs by decreasing the ability of the mutant enzyme to carry out excision. Mutations that map in the palm domain far from the PPi-binding site presumably use an indirect mechanism to confer PFA resistance, AZT hypersensitivity, and reduced excision activity, but the overall effect is the same. Binding of the RT·P/T binary complex to PFA is inhibited and ability to carry out excision is decreased.

## 4. Additional Mutations of HIV-1 RT that Suppress AZT Resistance (L74V, Y181C, L100I, V75I, M184V and K65R)

Suppression of AZT resistance mutations was initially observed for L74V, a mutation selected by ddI therapy [91]. This mutation reduces ATP-dependent excision in various TAM backgrounds [92]. Reduced excision below wild type level was also reported in the absence of TAMs (resulting in AZT hypersensitivity) [93]. Mutations Y181C and L100I, selected by NNRTI therapy, were also identified as suppressors of AZT resistance [94,95]. Y181C was associated with reduced ATP-dependent excision and reduced affinity of the RT·P/T binary complex for ATP [65]. Recently, it has been shown that V75I, which is selected as part of the Q151M multidrug resistance complex [for review, see Ref. 17], suppresses both AZT and d4T resistance in culture assays and leads to a substantial decrease in ATP-dependent excision activity in the context of a high-excision finger insertion mutant of RT [96]. V75I is only rarely associated with TAMs and the antagonistic biochemical relationship may help to explain that.

The suppression of AZT resistance by M184V has played an important role in the clinical use of NRTIs for antiretroviral therapy over the past 15 years. M184V was first identified due to its selection by 3TC therapy [97–100] and was subsequently shown to confer high-level resistance to FTC as well [101]. The mechanism of M184V resistance to 3TC and FTC involves increased ability of the mutant RT to distinguish between these analogs and the natural nucleotide, dCTP [17,102–104]. Soon after its discovery, M184V was recognized as a potent suppressor of AZT resistance conferred by TAMs [99–101]. This suppression is likely to play an important role in the beneficial effects of AZT-3TC combination therapy [105,106] although other factors such as the reduced fitness of virus containing the M184V mutation may also contribute. Suppressive effects of M184V on TAM-mediated resistance to d4T and TFV have been also been reported [107–109].

Biochemical effects of the M184V mutation on ATP-dependent excision by mutant RTs have been the subject of several studies [92,93,110–115]. Most investigations have shown that addition of M184V to TAM-containing RTs reduced ATP-dependent excision activity; however, the reports are not entirely consistent. Naeger *et al.* [111] reported no effect of M184V on excision activity, and Boyer *et al.* [114] reported that M184V reduced AZTMP excision when the assay mixture contained 100 μM dNTPs but not when 10 μM dNTPs were present. Various factors have been suggested to account for these inconsistencies including differences in primer-template sequence context and the presence of different combinations of TAMs; however, even in studies where decrease in the rate of excision is observed, the level of reduction is hard to reconcile with the potent suppressor phenotype observed for M184V in infectivity assays. As a result, the current mechanistic understanding of M184V suppression of TAMs is unsatisfying and other factors may remain to be defined.

As summarized in Table 1, K65R is selected by several NRTIs including ABC [116,117], TFV [118], d4T [119] and ddI [120], and confers resistance through a discrimination mechanism [86,121–123]. Discrimination between AZT and dTTP is also increased by K65R [78,122,123], but this is counteracted by reduction of ATP-dependent excision [37,78,115,122,123]. Suppression of AZT resistance may explain why K65R is rarely observed in combination with TAMs [37,78–80]. In the WT RT structure, K65 forms a salt bridge with the γ-phosphate of the incoming dNTP. The change from K to R increases the length of the side chain forming the salt bridge and alters the positioning of adjacent residues. This reduces the mobility of the loop structure in the fingers domain and impedes the conformational changes preceding catalysis [78,86]. Recently published structures [86] of ternary complexes containing K65R mutant RT, dsDNA primer-template, and TFV-DP or dATP, provide insight into the mechanisms by which K65R confers TFV resistance and reduces NRTI incorporation and excision. In these structures, the planar guanidinium moiety of R65 stacks with the guanidinium of R72 to form a platform introducing rigidity into the structure surrounding the active site. This disfavors the conformational change that rotates the fingers into the active site and reduces polymerase activity. The stacked guanidinium groups interact differently with TFV-DP than with dATP leading to more restricted movements of R72 in the K65R RT·P/T·TFV-DP complex than in the K65R RT·P/T·dATP complex. This provides a rationale for the discrimination by this mutant against TFV-DP. Increased fidelity of K65R RT [124,125] may also be explained by the decreased flexibility of the active site imposed by the R65–R72 stacking interaction. Decreased flexibility of the fingers loop subdomain of K65R RT is also invoked to explain reduced excision activity of this mutant since movement of this domain contributes to the ability of the β- and γ-phosphates of ATP to act as acceptor in the excision reaction [86]. The authors suggest that the effect on excision may be greater when TAMs are present since the R65–R72 platform could interact with TAM residues K70R and/or T215Y, limiting movement that is needed to accommodate ATP as excision substrate and dinucleoside tetraphosphate as excision product. Excision would be inhibited, for example, by restricting the π-π interactions between Y215 and the adenine moiety in ATP.

In summary for this section, mutations in RT that increase discrimination between chain-terminating analogs and natural substrates may also suppress AZT resistance. In addition, several of these mutations exhibit increased fidelity for dNTP incorporation during DNA synthesis [124,125]. The structural data for K65R RT suggest that these properties may derive primarily from the reduced flexibility in structures around the mutant polymerase active site due to the stable stacking interaction between the mutated K65R residue and R72. The phenotypes of other suppressor mutations may have similar explanations, but specific structural alterations have not been identified.

## 5. Indirect Enhancement of Excision due to Mutations in the RNase H and Connection Domains of RT

In 2004, the Pathak laboratory [126] reported that mutations H539N and D549N introduced into the RNase H domain of RT decreased the frequency of RT template switching resulting in reduced recombination frequency. Subsequently, these authors [127] showed that these mutations also conferred resistance to AZT, d4T and ddI. Because of these results, they proposed that a balance exists between RNase H activity and nucleotide excision *in vivo* and that reduced RNase H cleavage of the RNA template near the primer terminus could delay dissociation of the terminated DNA chain from the remaining template fragment allowing RT more time to carry out excision and remove the chain-terminating nucleotide. This hypothesis spurred numerous investigators to re-evaluate possible association between NRTI resistance and mutations in the C-terminal half of the RT coding region including the RNase H domain (codons 441–560) and the connection domain (codons 322–440). The importance of mutations in these domains has been confirmed in multiple studies [see reviews, Refs. 128–130]*,* and the hypothesis is also supported by *in vitro* studies [131–136]. For a more detailed discussion of NRTI resistance due to RNase H and connection domain mutations in RT see the review by Pathak *et al*. [137] in this special issue of *Viruses*.

## 6. Conclusions

Enhanced nucleotide-dependent excision is a major mechanism of AZT resistance by mutants of HIV-1 and these mutations confer some degree of resistance to most NRTIs. In the excision reaction, a chain-terminating residue is removed after incorporation, by transfer to a nucleoside triphosphate (likely, ATP in most infected cells). Mutations in RT that enhance excision act primarily on the catalytic rate and also stimulate the reverse reaction (incorporation of the ddNMP moiety from a dinucleoside tetraphosphate), suggesting that they facilitate the formation of a reaction intermediate that is rate-limiting for the reaction in both directions. Mutations that decrease the rate of nucleotide excision and suppress AZT resistance may act through various mechanisms. New insight into decreased excision conferred by the K65R mutation has been provided by crystal structures of the ternary complexes K65R RT·P/T·TFV-DP and K65R RT·P/T·dATP, which reveal a stable guanidinium stacking interaction between the mutated residue and R72 that restricts the conformational mobility of adjacent structures. Decreased mobility of the fingers loop region of RT is expected to reduce the efficiency of excision. Excision is also inhibited by indirect mechanisms that cause RT to preferentially occupy the post-translocation position since RT cannot carry out excision when bound in this position. For example, excision activity is inhibited by binding the next complementary dNTP, which locks RT in the post-translocation position, and by introduction of the E89K mutation, which interferes with RT binding in pre-translocation position. Excision is enhanced when AZT is at the primer terminus since the AZT structure favors RT binding in the pre-translocation position and disfavors binding of the next complementary dNTP. On an RNA template, excision can also be inhibited by RNase H cleavage near the primer terminus, which promotes dissociation of the remaining primer-template duplex. Excision is stimulated by RNase H mutants that decrease the frequency of these cleavages and prevent primer-template dissociation. Ultimately, the development of better therapies involving NRTIs may depend on our ability to elucidate in molecular detail the factors that influence excision rescue of DNA chains terminated with these drugs.

## Figures and Tables

**Table 1.** NRTIs currently used in clinical therapy ^a^.

**Common name**	**Abbrev.**	**Structural name**	**Natural analog**	**Year FDA approved**	**Common resistance mutations selected ^b^**	**Mechanism of resistance**
zidovudine	ZDV, AZT	β-d-(+)-3′-azido-3′-deoxythymidine	dTTP	1987	M41L, D67N, K70R, L210W, T215F/Y, K219Q/E	excision
didanosine	ddI	β-d-(+)-2′,3-dideoxyinosine	dATP	1991	K65R, L74V	discrimination
stavudine	d4T	β-d-(+)-2′,3′-didehydro-3′-deoxythymidine	dTTP	1994	M41L, D67N, K70R, L210W, T215F/Y, K219Q/E	excision
lamivudine	3TC	β-l-(−)-2′,3′-dideoxy-3′-thiacytidine	dCTP	1995	K65R, M184V/I	discrimination
abacavir	ABC	(1*S*,4*R*)-4-[2-amino-6-(cyclopropyl-amino)-9H-purin-9-yl]-2-cyclopentene-1-methanol succinate	dGTP	1998	K65R, L74V, Y115F, M184V	discrimination
tenofovir^c^	TFV	({[(2*R*)-1-(6-amino-9*H*-purin-9-yl)propan-2-yl]oxy}methyl)phosphonic acid	dATP	2001	K65R, K70E	discrimination
emtricitabine	FTC	β-l-(−)-2′,3′-dideoxy-5-fluoro-3′-thiacytidine	dCTP	2003	K65R, M184V/I	discrimination

a All NRTIs shown are approved by the U.S. Food and Drug Administration (FDA). Zalcitabine (ddC), which is not currently in use, is also FDA approved;

b From the current listing of mutations associated with antiretroviral drug resistance compiled by the International AIDS Society—USA, Drug Resistance Mutations Group [19];

c Prodrug is tenofovir disoproxil fumarate (TDF).

**Table 2.** PFA-resistant mutants of HIV-1 RT.

**Location in RT structure**	**How isolated?**	**Mutations**	**AZT sensitivity**	**Excision activity *in vitro***
dNTP binding site	Site-directed mutagenesis or bacterial library mutagenesis	D113G/E, A114S/G, Y115N/H, Q151H [66,70] R72A [71]	Hypersensitivity shown for D113E & A114S [70]	Reduced for A114S [72]
	From patients treated with NRTIs	K65R [73–76]	Suppression of AZT resistance [37,77–80]	Reduced for K65R [37,78]
Palm domain between the fingers domain and the template strand	From PFA-treated patients	W88S, W88G, Q161L, H208Y [68]	Suppression of AZT resistance shown for W88G and Q161L [37] No effect for W88S [37]	Reduced for W88G and Q161L [37] Not reduced for W88S [37]
	Serial passage of HIV-1 in culture	Q161L/H208Y [68] E89K, L92I, S156A [67] W88G, S117T, F160Y, M164I [76]	Suppression of AZT resistance shown for W88G, E89K, Q161L/H208Y, S117T, and M164I [37,67–69]	Reduced for W88G, E89K, Q161L/H208Y, S117T, and M164I [37]
	Site-directed or random mutagenesis	K154E, Y183S, G190R [66,70,81] E89G/T/A/D/V[82,83] V90A [84]	AZT sensitivity not determined in infectivity assays	Not done
